# Health facility delivery in sub-Saharan Africa: successes, challenges, and implications for the 2030 development agenda

**DOI:** 10.1186/s12889-018-5695-z

**Published:** 2018-06-19

**Authors:** Henry V. Doctor, Sangwani Nkhana-Salimu, Maryam Abdulsalam-Anibilowo

**Affiliations:** 1 World Health Organization (WHO), 20 Avenue Appia, CH-1211 Geneva, Switzerland; 2 World Health Organization (WHO) Regional Office for the Eastern Mediterranean, Cairo, Egypt; 3 College of Medicine, University of Malawi, Blantyre, Malawi; 4 Institute of Human Virology, Abuja, Nigeria

**Keywords:** Health facility birth, Maternal mortality, Neonatal mortality, Skilled birth attendants, Sub-Saharan Africa

## Abstract

**Background:**

Sub-Saharan Africa remains one of the regions with modest health outcomes; and evidenced by high maternal mortality ratios and under-5 mortality rates. There are complications that occur during and following pregnancy and childbirth that can contribute to maternal deaths; most of which are preventable or treatable. Evidence shows that early and regular attendance of antenatal care and delivery in a health facility under the supervision of trained personnel is associated with improved maternal health outcomes. The aim of this study is to assess changes in and determinants of health facility delivery using nationally representative surveys in sub-Saharan Africa. This study also seeks to present renewed evidence on the determinants of health facility delivery within the context of the Agenda for Sustainable Development to generate evidence-based decision making and enable deployment of targeted interventions to improve health facility delivery and maternal and child health outcomes.

**Methods:**

We used pooled data from 58 Demographic and Health Surveys (DHS) conducted between 1990 and 2015 in 29 sub-Saharan African countries. This yielded a total of 1.1 million births occurring in the 5 years preceding the surveys. Descriptive statistics were used to describe the counts and proportions of women who delivered by place of delivery and their background characteristics at the time of delivery. We used multilevel logistic regression model to estimate the magnitude of association in the form of odds ratios between place of delivery and the predictors.

**Results:**

Results show that births among women in the richest wealth quintile were 68% more likely to occur in health facilities than births among women in the lowest wealth quintile. Women with at least primary education were twice more likely to give birth in facilities than women with no formal education. Births from more recent surveys conducted since 2010 were 85% more likely to occur in facilities than births reported in earliest (1990s) surveys. Overall, the proportion of births occurring in facilities was 2% higher than would be expected; and varies by country and sub-Saharan African region.

**Conclusions:**

Proven interventions to increase health facility delivery should focus on addressing inequities associated with maternal education, women empowerment, increased access to health facilities as well as narrowing the gap between the rural and the urban areas. We further discuss these results within the agenda of leaving no one behind by 2030.

## Background

Maternal mortality is one of the key health challenges in developing countries and sub-Saharan Africa in particular [1]. According to estimates in 2015, there were 303,000 maternal deaths with most of them occurring due to complications related to pregnancy and childbirth. Almost all of the 303,000 deaths occurred in low-resource settings such as sub-Saharan Africa [2]; and most of these deaths could be prevented. The good news is that between 1990 and 2015, maternal mortality worldwide dropped by about 44%, but this is low compared to the target set by the Millennium Development Goal (MDG) 5 to reduce maternal mortality worldwide by 75% by 2015. Therefore, as part of the Sustainable Development Goal (SDG) 3 on health, the target is to reduce the global maternal mortality ratio (MMR) to less than 70 deaths per 100,000 live births [1].

There are complications that occur during and following pregnancy and childbirth that can contribute to maternal deaths. Most of these complications are preventable or treatable. More than half of maternal deaths take place within one day of birth [3]. Malnutrition, including iodine deficiency, maternal anaemia, and poor-quality diet, also contribute to maternal mortality and the high incidence of stillbirths [3]. Mothers who are HIV positive are also 10 times more likely to die than mothers who are HIV negative [3]. According to the World Health Organization, most maternal deaths in sub-Saharan Africa are related to direct obstetric complications mainly haemorrhage, hypertension, sepsis, and obstructed labour, which combined account for 64% of all maternal deaths [4]. Pneumonia and HIV/AIDS account for 23%, and unsafe abortion accounts for 4% of maternal deaths in Africa [4].

The link between early and regular attendance of antenatal care and health facility delivery and improved maternal health outcomes has been documented for a considerable time. However, at least half of all births in developing countries occur in the absence of skilled birth attendants. This is largely influenced by socio-cultural factors, lack of understanding on the importance of skilled attendance at birth, financial hardship and physical accessibility [5]. According to Gabrysch and Campbell [5], socio-cultural factors often affect the decision to seek care compared to whether women actually reach the health facility. With respect to perceived benefit/need, the influence on delivery with skilled attendance is associated with factors related to women’s perception of the benefit of skilled attendance towards their health including that of their newborns. Economic accessibility refers to the ability of the family to meet the financial and transportation costs associated with the facility delivery. Physical accessibility indirectly affects decision-making to seek care and the ability to access health services after reaching a facility [5]. These challenges have made it difficult to achieve the MDG of global reduction of maternal deaths. The role of health facility delivery in improving maternal and child health cannot be overemphasized; and it is one of the key stepping stones towards achieving the SDG 3.

Health is at the epicentre of the post-2015 development agenda. In particular, the 2030 Agenda for Sustainable Development seeks to achieve integrated goals and targets related to social, environmental, and economic factors. In order to address gaps in health care delivery, Universal Health Coverage (UHC) was included as target 3.8 as part of SDG 3. Specifically, SDG 3.8 aims at achieving UHC, including financial risk protection by improving access and quality of healthcare delivery including improved access to safe, effective, quality and affordable essential medicines and vaccines for all individuals. Therefore, under SDG 3, the global MMR is expected to reach under 70 deaths per 100,000 live births by 2030. Therefore, this study not only uses a rich source of data from sub-Saharan Africa but also builds on target 3.8 to assess changes in and determinants of health facility delivery from nationally representative surveys in sub-Saharan Africa. The study also seeks to present renewed evidence within the context of the SDG agenda to generate data for evidence-based decision making and enable deployment of targeted interventions to improve maternal health outcomes.

## Methods

### Data sources

We use data from Demographic and Health Surveys (DHS) conducted between 1990 and 2015 in 29 sub-Saharan African countries. The surveys are grouped into two: “earliest” surveys conducted since 1990 (before the onset of the MDG agenda) and “latest” or most recent surveys conducted since 2010 but before 2015, close to the MDG deadline of 2015. By implication, countries which had only one DHS during this period were not included in the analysis. The time interval between the earliest and most recent DHS data provides sufficient time to observe reasonable changes in health facility delivery between the period before the MDG agenda and the period close to the MDG deadline. A total of 24 surveys (from 12 countries) come from Western Africa; 8 surveys (from 4 countries) come from Middle Africa; 22 surveys (from 11 countries) come from Eastern Africa; and 4 surveys (from 2 countries) come from Southern Africa (Table 1). Time intervals between the earliest and latest surveys ranged between 5 and 23 years, averaging 15 years of observation. The pooled DHS data include 396,837 births from earliest surveys and 762,445 from latest surveys; yielding a total of 1.1 million births occurring in the 5 years preceding the surveys. The pooled data set was based on birth history files where each woman was asked for the date of birth (month and year) of each live-born child, the child’s sex, whether the child was still alive (and if the child had died) the age at death (in days for the first month, in months if the deaths occurred between 1 and 24 months, and in years thereafter). These data allowed child deaths to be located by time and by age.

**Table 1:** Countries and Demographic and Health Surveys included in the analysis for 29 sub-Saharan African countries

Country	Earliest Survey	Latest Survey	Observation time^a^
Western Africa (*n* = 12)			
Benin	1996	2011–2012	16
Burkina Faso	1993	2010	17
Cote d’Ivoire	1994	2011–12	18
Ghana	1993	2014	21
Guinea	1999	2012	13
Liberia	2007	2013	6
Mali	1995–96	2012–13	17
Niger	1998	2012	14
Nigeria	1990	2013	23
Senegal	1997	2014	17
Sierra Leone	2008	2013	5
Togo	1998	2013–14	16
Middle Africa (*n* = 4)			
Cameroon	1991	2011	20
Congo (Brazzaville)	2005	2011–12	7
Congo Democratic Republic	2007	2013–14	7
Gabon	2000	2012	12
Eastern Africa (*n* = 11)			
Comoros	1996	2012	16
Ethiopia	2000	2011	11
Kenya	1993	2014	21
Madagascar	1997	2008–09	12
Malawi	1992	2010	18
Mozambique	1997	2011	14
Rwanda	1992	2010	18
Tanzania	1996	2010	14
Uganda	1995	2011	16
Zambia	1996	2013–14	18
Zimbabwe	1994	2010–2011	17
Southern Africa (*n* = 2)			
Lesotho	2004	2014	10
Namibia	1992	2013	21
Summary statistics			
Minimum observation time (years)		5	
Maximum observation time (years)		23	
Mean observation time (years)		15	
Standard deviation		4.7	
Lower and upper quartiles (years)		[6, 20]	

Notes: ^a^Observation time calculated based on the upper bound of the year. For example, the 2010–2011 year uses 2011 as the end point. Latest surveys defined as those from 2010 with the exception of Madagascar (2008–09)

Source: [22]

### Statistical analysis

We performed statistical analysis using Stata version 14 [6]. We used descriptive statistics to describe the counts and proportions of women who delivered by place of delivery and their background characteristics at the time of delivery. The reference event for all analyses were most recent birth during the 5 years preceding the surveys. We consider the following predictors of place of delivery: wealth status ranking based on wealth quintiles; residence (urban/rural); mother’s characteristics (education, having at least one antenatal care (ANC) visit, age of mother at birth); community women’s education (none or at least primary education); birth order of child; and a dummy indicator for the survey round (earliest/latest). Place of delivery was coded as ‘1’ for children who were born in a health facility and ‘0’ for children who were delivered elsewhere (Table 2). The percent of missing data for the variables concerned ranges from 1.2 to 4.9% and these were excluded from the analyses.

**Table 2:** Variables used in the analysis of predictors of place of delivery among women with most recent births for 29 sub-Saharan African countries

Variable	Coding categories	Description/definitions
Dependent variable		
Place of delivery	0: Non-health facility (Ref); 1: Health facility	Place where the woman delivered
Independent variables		
Wealth quintile	1: Lowest (Ref); 2: Second; 3: Third; 4: Fourth; 5: Highest	Measure of household wealth status based on household assets
Residence	1: Rural (Ref.); 2: Urban	Urban or rural residence
Education level	1: None (Ref.); 2: At least primary	Highest education level attained by the respondent
Community women’s education	1: Low (Ref.); 2: Medium; 3: High	Community level education measured as the proportion of women with at least primary education in the primary sampling unit. The measure was divided into 3 tertiles and categorized as low, medium and high.
Age at birth	1: < 20 (Ref.); 2: 20–24; 3: 25–29; 4: 30+	Mother’s age at birth
Birth order	1: 1 (Ref); 2: 2–3; 3: 4+	Birth order of child for most recent birth
Round of survey period	1: Earliest (Ref); 2: Latest	Round of survey period for the 29 countries
Region	1: Western Africa (Ref); 2: Middle Africa; 3: Eastern Africa; 4: Southern Africa	Sub-Saharan African region (see country list in Table 1)

Note: “Ref.” – Reference category

We used multilevel logistic regression model to estimate the magnitude of association in the form of odds ratios (ORs) between place of delivery and the predictors. In particular, multilevel models were constructed using the mixed effects modelling procedure where data have been collected in nested units. Sampling cluster was included in the model as nested random effects with country modelled as fixed effects. For the purposes of the analysis, we fit unadjusted regression models for each explanatory variable and then fit two additional models: **Model 0** (empty model) excludes independent variables in order to decompose the total variance into its cluster and country components. **Model 1** is the full model which includes all independent variables.

The three-level multi-level model to estimate the cluster and country effects is written as follows, eq. (1):

1 $$ \kern0.5em logit\ \left({\pi}_{ijk}\right)=\log \left(\frac{\pi_{ijk}}{1-{\pi}_{ijk}}\right)={\beta}_0+{X}_{ijk}+{u}_{0 jk}+{v}_{0k} $$

where *π*_*ij*_ is the probability that the *i*th woman of *j*th cluster in the *k*th country will deliver in a health facility; *X*_*ij*_ is a set of variables for each *i*th woman of the *j*th cluster in the *k*th country. These covariates may be defined at the individual, community, or country level; *β*_0_ is the associated vector of standard regression parameter estimates; *u*_0*jk*_ represents the random effect at the cluster level; and *v*_0*k*_ is the random effect at the country level. The intercept or average probability of a woman delivering in a health facility is assumed to vary randomly across clusters and countries. Based on this approach, the fixed effects (measures of association) are presented as odds ratios (OR) alongside 95% confidence intervals (CI). We tested the goodness of fit of the multilevel model using the log likelihood ratio (LR) test.

This approach led to estimation of unadjusted and adjusted ORs of the likelihood of health facility delivery. Independent variables were included if they were statistically significantly associated with the outcome variable with a cut-off *p*-value of < 0.05 in the multilevel logistic regression model. The adjusted ORs were an outcome of the multilevel logistic regression (i.e. eq. (1)) in estimating the net contribution of each covariate to the outcome variable, adjusting for other covariates in the model. An OR of 1 implied no difference whereas an OR > 1 implied the woman was more likely to deliver in a health facility; and an OR < 1 implied less likelihood of a woman delivering in a health facility. All statistical tests were set at 5% level of significance with associated 95% confidence intervals.

The adjusted ORs of place of delivery from the multilevel logistic regression model for each country were used to conduct meta-analysis in Stata to develop a forest plot of the adjusted pooled effect (i.e. women who delivered the most recent child in a health facility compared to women who delivered elsewhere) across 29 countries. The pooled effect focuses on health facility delivery during latest survey rounds compared with earliest survey round. The pooled ORs with associated 95% confidence intervals (CI) were estimated using Mantel-Haenszel statistical methods. Heterogeneity among the surveys was assessed using *I*^*2*^ statistics, a measure of the proportion of total variability explained by heterogeneity rather than chance expressed as a percentage [7]. Roughly, an *I*^*2*^ of 0–40% represents no or little heterogeneity, 30–60% moderate heterogeneity, 50–90% substantial heterogeneity, and 75–100% considerable heterogeneity [8]. The meta-analysis applied random effects analytical model due to the considerable heterogeneity (> 75%) among the survey results. Observed likelihood of delivering in a health facility were compared with expected likelihood of health facility delivery which were obtained after adjusting for the risk factors in the regression model.

Independent variables were subjected to multi-collinearity tests by performing correlations, variable inflation factor (VIF) and tolerance tests. The mean VIF was 1.43 whereas tolerance values were at least 0.5 [9]. The VIF between several variables that potentially had multicollinearity such as mother’s education, community women’s education, and wealth quintile were also at least 0.5; and these tests indicated no cause for concern for collinearity. We applied sample weights for descriptive analyses using the Stata svy command to account for undercounting and over counting due to the sample design of the survey [6].

## Results

### Descriptive findings

Table 3 shows the weighted number and percentage distribution of all women by place of delivery and background characteristics. By quintile, the majority of births (22.9%) occurred among women from households belonging to the lowest quintile. About 7 out of 10 births (73.3%) of births took place in rural areas; with similar number of births occurring among women with no schooling and those with at least primary schooling. There were almost the same number of births among women across the categories of community women’s education. More births occurred among women with who did not receive antenatal care (ANC; 78.5%), aged 20–24 years (31.9%), from the latest survey period (65.1%), and from Eastern Africa (37.8%).

**Table 3:** Weighted number and percentage distribution of women by place of delivery and background characteristics, Demographic and Health Surveys, 29 sub-Saharan African countries

Background characteristics	Health facility	Non-health facility	Total
	Number (%)	Number (%)	Number (%)
Wealth quintile***			
Lowest	39,665 (15.0)	225,440 (85.0)	265,105 (22.9)
Second	47,627 (18.5)	210,356 (81.5)	257,983 (22.3)
Third	52,835 (21.8)	189,115 (78.2)	241,950 (20.9)
Fourth	59,969 (27.2)	160,429 (72.9)	220,408 (19.0)
Highest	62,382 (35.9)	111,286 (64.1)	173,668 (15.0)
Residence***			
Rural	155,605 (17.6)	727,360 (82.4)	882,975 (73.3)
Urban	113,019 (35.1)	209,155 (64.9)	322,174 (26.7)
Woman characteristics			
Mother’s education***			
None	82,615 (13.6)	525,477 (86.4)	608,092 (50.5)
At least primary	185,986 (31.2)	410,937 (68.8)	596,923 (49.5)
Community characteristics			
Community women’s education***			
Low	80,135 (19.8)	324,523 (80.2)	404,658 (33.6)
Medium	89,403 (21.8)	321,021 (78.2)	410,424 (34.1)
High	99,086 (25.4)	290,970 (74.6)	390,057 (32.4)
Pregnancy characteristics			
Number of antenatal care (ANC) visits***			
None	90,545 (9.6)	854,939 (90.4)	945,483 (78.5)
At least once	177,104 (68.6)	81,345 (31.4)	259,249 (21.5)
Mother’s age at birth***			
< 20	43,840 (15.6)	236,971 (84.4)	280,812 (23.3)
20–24	78,008 (20.3)	306,779 (79.3)	384,787 (31.9)
25–29	68,317 (24.3)	213,063 (75.7)	281,380 (23.3)
30+	78,458 (30.4)	179,679 (69.6)	258,137 (21.8)
Child-specific characteristics			
Birth order***			
1	73,901 (22.5)	253,929 (77.5)	327,830 (27.2)
2–3	99,585 (22.1)	351,579 (77.9)	451,164 (37.4)
4+	95,138 (22.3)	331,006 (77.7)	426,144 (35.4)
Survey characteristics			
Round of survey period***			
Earliest	59,847 (15.8)	319,119 (84.2)	378,966 (34.9)
Latest	173,885 (24.6)	534,258 (75.4)	708,143 (65.1)
Region***			
Western Africa	99,814 (19.4)	415,395 (80.6)	515,209 (47.9)
Middle Africa	41,846 (32.8)	85,656 (67.2)	127,502 (11.9)
Eastern Africa	82,512 (20.3)	324,165 (79.7)	406,678 (37.8)
Southern Africa	7687 (30.1)	17,842 (69.9)	25,530 (2.4)
Total	268,624 (22.3)	936,515 (77.7)	1,205,139 (100.0)

Note: ***p < 0.001

Table 3 also shows that with respect to wealth quintile, the highest percentage of births occurring in health facility were among women in the highest quintile (35.9%). Of all births occurring in rural areas, 17.6% occurred in a health facility compared with 35.1% of all urban births that took place in a health facility. Almost 14% of births of mothers with no education occurred in health facility compared with 31.2% of births among women with at least primary education. More births (25.4%) occurred in health facilities among women living in communities with a high proportion of mothers with at least primary schooling compared with 21.8 and 19.8% of births occurring among women living in communities with medium and low concentration of mothers with at least primary education. At least two-thirds (68.6%) of mothers who had at least one ANC visit delivered in a health facility compared with 9.6% of births whose mothers received no ANC. Older mothers (30+ years) reported a higher percentage (30.4) of births occurring in a health facility than younger mothers. There were no differences in facility delivery by birth order. Slightly more births (32.8%) occurred in health facilities in Middle Africa than in the other sub-Saharan African regions. More health facility births occurred during the latest survey years (24.6%) than during the earliest survey period (15.8%). Overall, out of the 1.2 million births that occurred among women aged 15–49 years in the 29 sub-Saharan African countries during the earliest and latest surveys, 268,624 (22.3%) births occurred in a health facility and 966,515 (77.7%) births occurred outside health facilities.

### Multivariate logistic regression results

Multilevel logistic regression results are presented in Table 4. Unadjusted ORs show that the ORs of health facility deliveries increased by wealth quintile, ranging from 1.25 (95% C.I: 1.23–1.27) in the second quintile to 2.84 (95% C.I: 2.78–2.89). Thus, the likelihood of health facility delivery was higher in all wealth quintiles compared with the lowest quintile. Women in urban areas were 2.66 times more likely to deliver their babies in health facilities (OR: 2.66; 95% C.I: 2.61–2.70) than women in rural areas. Births among women with at least primary schooling were 2.46 times more likely (OR: 2.46; 95% C.I: 2.43–2.49) to occur in health facilities than births among women with no schooling. Women living in communities with medium and high levels of community women’s education were associated with higher odds of health facility births (OR: 1.16; 95% C.I: 1.12–1.20) and (OR: 1.33; 95% C.I: 1.29–1.38), respectively, than women living in communities with low levels of community women’s education. Women with at least one ANC visit were more likely to deliver their children in health facility compared with women who did not receive ANC (OR: 25.78; 95% C.I: 25.44–26.13). Women aged at least 20 years were more likely to report their births delivered in a health facility than women aged under 20 years; with ORs range from 1.29 for the 20–24 year age group to 2.70 among women aged 30 years and older. Children of birth order 2 or 3 were 9% more likely (OR: 1.09; 95% C.I: 1.08–1.10) to be delivered in the health facility than children of birth order 1; and children of birth order 4 and above were 47% more likely (OR: 1.47; 95% C.I: 1.45–1.48) to be delivered in the health facility than children of birth order 1. By the latest surveys, births were 60% more likely to be delivered in a health facility than births during the earliest surveys (OR: 1.60; 95% CI: 1.58–1.62). The only significant result for sub-Saharan African region shows that births from Middle Africa were 2.04 (95% C.I: 1.16–3.57) times more likely to be delivered in a health facility than births from Western Africa.

**Table 4:** Unadjusted and adjusted multilevel logistic regression of a woman giving birth in a health facility by predictor variables for 29 sub-Saharan African countries

Determinants	Unadjusted OR (95% CI)	Model 0 (Empty Model)	Model 1 Adjusted OR (95% CI)^a^
Fixed effects			
Household characteristics			
Wealth quintile			
Lowest	Ref		Ref
Second	1.25 (1.23–1.27)***		1.18 (1.15–1.20)***
Third	1.48 (1.45–1.50)***		1.29 (1.27–1.32)***
Fourth	1.86 (1.83–1.90)***		1.43 (1.40–1.47)***
Highest	2.84 (2.78–2.89)***		1.68 (1.63–1.72)***
Residence			
Rural	Ref		Ref
Urban	2.66 (2.61–2.70)***		2.02 (1.97–2.06)***
Woman characteristics			
Mother’s education			
None	Ref		Ref
At least primary	2.46 (2.43–2.49)***		1.84 (1.82–1.88)***
Community characteristics			
Community women’s education			
Low	Ref		Ref
Medium	1.16 (1.12–1.20)***		1.08 (1.04–1.11)***
High	1.33 (1.29–1.38)***		1.11 (1.08–1.15)***
Pregnancy characteristics			
Number of ANC visits			
None	Ref		Ref
At least once	25.78 (25.44–26.13)***		23.71 (23.38–24.04)***
Mother’s age at birth			
< 20	Ref		Ref
20–24	1.29 (1.27–1.31)***		1.35 (1.33–1.38)***
25–29	1.70 (1.67–1.72)***		1.74 (1.70–1.78)***
30+	2.70 (2.67–2.74)***		2.37 (2.31–2.44)***
Child-specific characteristics			
Birth order			
1	Ref		Ref
2–3	1.09 (1.08–1.10)***		0.75 (0.73–0.76)***
4+	1.47 (1.45–1.48)***		0.62 (0.60–0.63)***
Survey characteristics			
Survey round			
Earliest	Ref		Ref
Latest	1.60 (1.58–1.62)***		1.85 (1.81–1.88)***
Region			
Western Africa	Ref		Ref
Middle Africa	2.04 (1.16–3.57)**		1.54 (0.85–2.82)
Eastern Africa	0.99 (0.66–1.49)		0.78 (0.51–1.21)
Southern Africa	1.56 (0.74–3.27)		1.05 (0.48–2.33)
Random effects			
*Country-level*			
Variance (SE)		0.557 (0.074)***	0.529 (0.070)***
Intra-country correlation (SE)		0.072 (0.018)	0.067 (0.016)
*Cluster-level*			
Variance (SE)		0.836 (0.006)	0.728 (0.006)***
Intra-cluster correlation (SE)		0.235 (0.015)	0.197 (0.014)
LR test vs. logistic model		96,382.99	51,425.27
Prob > Chi2		0.001	0.001

Notes: ANC – Antenatal care; CI – confidence interval; ***p < 0.001; SE – Standard error; **p < 0.05; Ref – Reference category; OR – odds ratio

^a^Odds ratios were calculated using unadjusted and adjusted multivariate analysis. A total of 1,159,282 births were included in the analysis

Multi-level regression results for the empty model (Model 0) in Table 4 show that the total variance in health facility delivery associated with country context was significant across the 29 countries (*τ*=0.557, *p* < 0.001). Similarly, the variance was significant across communities (*τ*=0.836, *p* < 0.001). The intra-country correlation was 7.2% indicating that there was variance in health facility delivery at the country level. The intra-cluster correlation was 23.5% indicating that there was variance in health facility delivery at the cluster level. A likelihood ratio (LR) test comparing the empty model to an ordinary logistic regression model was highly significant for the data.

Regression results adjusted for all the variables generally show the same direction of effect although the magnitude of some estimates is attenuated. In particular, the effect of birth order is reversed showing that births of higher order were less likely to be delivered in a health facility than first births. The effect of sub-Saharan African region also disappears in the full model.

For the adjusted Model 1, the total variance in health facility delivery associated with country context remained significant across all the 29 countries (*τ*=0.529, *p* < 0.001). Similarly, the variance was statistically significant across communities (*τ*=0.728, *p* < 0.001). The intra-country correlation was 6.7% indicating the presence of variance in health facility delivery at the country level. The intra-cluster correlation was 19.7% indicating the presence of variance in health facility delivery at the cluster level. Using a likelihood ratio (LR) test compare **Model 0** to an ordinary logistic regression model showed that the results were highly significant for the data.

**Fig. 1:**
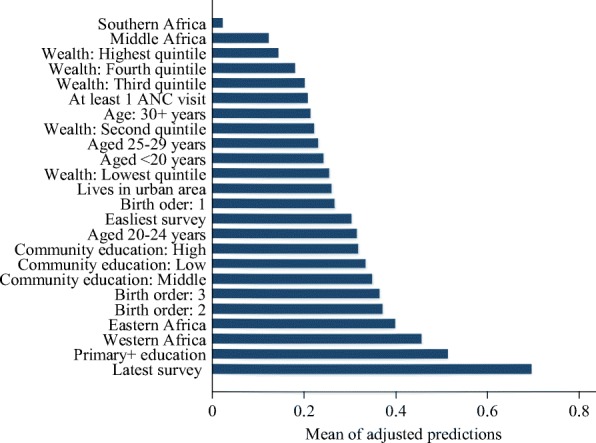
Adjusted predictions of the likelihood (odds ratio) of health facility delivery at the means of the independent variables for 29 sub-Saharan African countries

Figure 1 displays the likelihood of women reporting health facility births at the means of the covariates. In a hypothetical situation where all the other variables at set at their means or average values, the predicted likelihood of women reporting health facility births was highest among women interviewed during the latest surveys followed by women with at least primary education, women living in Western Africa, and women living in Eastern Africa.

In our analysis, we compared results from the observed health facility delivery with similar results from the post-estimation multilevel regression models to assess differences in the observed and predicted health facility delivery. In the adjusted model, the observed health facility delivery was 2% higher than the expected health facility delivery (i.e. 22.3 vs 21.9%). Across all respondents characteristics, the observed health facility delivery was 6% higher than expected (Ratio = 1.06) among respondents from communities with the highest percentage of women who completed at least primary schooling; with Southern African region having the highest observed health facility delivery higher than expected at 2% (Ratio = 1.02) (Table 5). Country-specific observed and expected health facility delivery rates are presented in Appendix.

**Table 5:** Observed and expected proportion of health facility delivery for 29 sub-Saharan African countries

Characteristics	Health facility delivery		Ratio (3) = (1) / (2)
	Observed (1)	Expected (2)	
Wealth index			
Lowest	15.0	14.3	1.05
Second	18.5	17.9	1.03
Third	21.8	20.9	1.04
Fourth	27.2	26.2	1.04
Highest	35.9	35.6	1.01
Residence			
Rural	17.6	17.2	1.02
Urban	35.1	35.2	1.00
Mother’s education			
None	13.6	13.4	1.01
At least primary	31.2	30.3	1.03
Community women’s education			
Low	19.8	20.0	0.99
Medium	21.8	22.0	0.99
High	25.4	23.9	1.06
Number of antenatal care visits			
None	9.6	9.6	1.00
At least once	68.6	68.3	1.00
Mother’s age at birth			
< 20	15.6	15.7	0.99
20–24	20.3	19.9	1.02
25–29	24.3	23.7	1.03
30+	30.4	30.1	1.01
Birth order			
1	22.5	22.0	1.02
2–3	22.1	21.4	1.03
4+	22.3	22.5	0.99
Round of survey period			
Earliest	15.8	16.2	0.98
Latest	24.6	24.6	1.00
Region			
Western Africa	19.4	19.8	0.98
Middle Africa	32.8	32.7	1.00
Eastern Africa	20.3	20.7	0.98
Southern Africa	30.1	29.4	1.02
All deliveries	22.3	21.9	1.02

### Meta-analysis of prevalence of health facility delivery

**Fig. 2:**
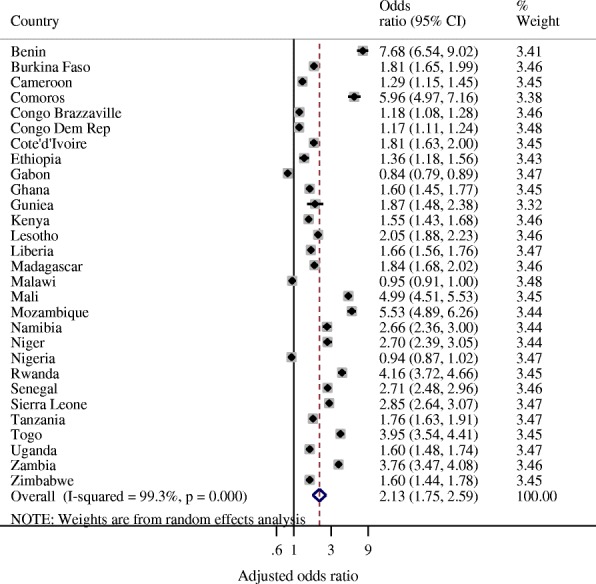
Forest plot of adjusted odds ratios of observing health facility delivery during latest survey rounds compared with earliest survey rounds for 29 sub-Saharan African countries (i.e. 58 surveys)

The overall meta-analysis (Fig. 2) of health facility delivery during latest survey round compared with earliest survey round includes 1,159,282 births for 29 countries and 58 surveys. That is, Fig. 2 displays the pooled adjusted ORs from multi-level logistic regression analyses for each country - similar to results presented in Table 4. The pooled adjusted OR demonstrated that women interviewed during the latest survey rounds were 2.13 times more likely to deliver in a health facility than women interviewed during earliest survey rounds (aOR = 2.13, 95% CI: 1.75–2.59). The results showed considerable heterogeneity between the most recent surveys (*I*^*2*^ = 99.3%). The weights correspond to the weights used to get the overall pooled adjusted OR.

## Discussion

Using data from 58 Demographic and Health Surveys from 29 sub-Saharan African countries, our study provides an opportunity to examine changes in health facility delivery as one of the components of health service delivery systems under the umbrella of Universal Health Coverage (UHC) [10]. Examining changes since 1990 provides an opportunity to understand the existing gaps and possible interventions to implement in order to improve maternal and health outcomes in sub-Saharan Africa by 2030. We found an overall increase in more births being delivered in health facilities in later surveys (conducted since 2010) compared to earlier surveys (conducted since 1990s).While this increase is news noteworthy, almost 40% of births are not attended by skilled personnel in sub-Saharan Africa compared with 96% of births in developed countries which are attended by skilled personnel [11]. Achieving the Sustainable Development Goal (SDG) 3 to reduce the MMR to less than 70 deaths per 100,000 live births by 2030 will require effective delivery and postpartum care to reduce preventable maternal and newborn deaths. This can be enhanced by health facility births under the care of skilled personnel.

Our study also found that the pattern of health facility deliveries varies within clusters or communities as well as within countries. While considering the fact that women from the same community will experience similar likelihood of delivering in a health facility, the results of this study highlight the importance of clustering effects in explaining differences in health facility delivery in sub-Saharan Africa. These effects are also observed at the country level. Building on the results from this study, available data shows that globally, births under the supervision of skilled personnel increased from 58% in 1990 to 78% in 2015 [12]; and this increase was influenced by increases in facility births in urban areas. We also found similar results across the 29 countries in our study: the odds of urban women delivering in a health facility more than doubled the odds of rural women delivering in a health facility. Possible contributing factors for low health facility births in rural areas have often been linked to key factors such as limited access and proximity to health centres, cost of health care services, female autonomy, time available to access health care [13] and myths about health facility delivery in some settings such as northern Nigeria [14]. This disparity negatively affects under-5 mortality rates and neonatal mortality rates at the national, regional and international level. Interventions targeting the reduction in inequalities in access to health care are pivotal towards improving maternal outcomes in sub-Saharan Africa. The importance of the interplay between maternal health outcomes and rural/urban disparities is also reported in several studies in sub-Saharan Africa [15, 16].

Our study also supports findings that maternal educational attainment and community women’s education are positively associated with health facility delivery. This finding further emphasizes the importance of interventions targeted at increasing women’s educational attainment. With increased maternal education, women are more likely to have more material resources and autonomy to access health care service [17, 18].

Other studies from sub-Saharan Africa have also confirmed that wealth is also closely related to place of delivery. That is, poorest women are least likely to use facility delivery services [16]. Our study provides further evidence towards this argument. Women from higher socio-economic status levels were more likely to deliver in health facilities than those from the lower socio-economic status levels.

With respect to children’s birth order, there is substantial evidence to suggest that facility delivery is more likely to decrease with the birth of the second or later children. However, insignificant differences are noted between second child and later births. A similar study in Nigeria suggests such trends may indicate that women of higher parity may stay away from health facilities due to increased maternal experiences or may be facing economic challenges due to increased family sizes, which may result to poor economic access to health facility [18]. A systematic review of studies in sub-Saharan Africa also links higher parity to lower likelihood of health facility delivery [16]. A systematic review of health financing policies in sub-Saharan Africa also documented varying degrees of policies that provide user fee exemption or reduction; national health insurance coverage; performance-based financing and user exemption; community insurance and other financing mechanisms that do not provide optimum health care services for families or women [19]. When children have higher birth orders, they may not benefit from the range of available services due to economic challenges.

This study also contributes to a body of literature on the relationship between ANC and facility based delivery. The findings are consistent with evidence and confirm the study hypotheses that ANC attendance is predictive of facility based delivery. In particular, a very significant difference exists between women who never utilised ANC services and those who did. Similar results are reported in Tanzania and Ghana [16] and Tanzania [15]. Further, the study in Tanzania attributed significant differences between two or more ANC visits and health facility delivery, especially in rural areas. The Tanzania study also found that one visit did not usually lead to facility based delivery. In bivariate analyses, our analyses found that at the regional level, women in Middle Africa were more likely to deliver in health facilities than women in Western Africa. However, this effect was no longer significant in the adjusted regression models which implies that the effect of region is not pronounced when other factors are taken into consideration.

In general, later surveys were more associated with health facility delivery than earlier ones. The overall ratio of the observed to expected facility births showed that observed facility births were only 2% more than what would be expected. This is a very low ratio and underscores that the observed increases in facility births are still too low to show a significant impact in improving maternal and child health outcomes. While proximity to health centers and lack of access have been highlighted as key contributors to global maternal mortality and subsequently neonatal and under-5 mortality rates, least developed countries such as those in sub-Saharan Africa are faced with persistent challenges such as substandard quality of care, poor sanitation and dwindling economic opportunities which slow down progress in improving health outcomes [14, 20]. Further cultural beliefs and norms such as gender inequity may be responsible for the observed low rates of health facility delivery in sub-Saharan Africa, in addition to challenges related to physical access. For example, women may be constrained from seeking health care services due to lack of permission from their spouses [20].

Proven interventions to improve maternal and newborn health can be implemented during labour, delivery and postpartum period. Among other things, these interventions relate to diagnosis and monitoring progress of labour; maternal and child health; detection and management of complications; delivery and immediate care of the newborn baby; breastfeeding and postnatal care. Treatment and management of any complications can also be provided to women who deliver in health facilities [21].

As the global community moves towards the deadline for achieving SDG 3 on health in 2030, countries are called upon to implement interventions aimed at achieving UHC. To achieve UHC, countries are called upon to strengthen health systems and implement robust health financing structures. In settings where out of pocket health expenditure is high, the poor are often disadvantaged and unable to access most of the health services. The rich may equally be disadvantaged particularly during severe or long-term illness. Recommended interventions also include pooling financial resources using compulsory mechanisms such as mandatory insurance schemes to defray the financial risks and promote good health among people. With reference to health facility delivery, UHC can be achieved by improving the capacity and availability of the healthcare workforce to deliver high quality services to people through integrated care [10]. Investing in healthcare workforce is inevitable in order to address inequities in access to healthcare. Involving rural and disadvantaged communities in programming and delivery of interventions to improve health outcomes can lead to significant increases in health facility delivery [20] and accelerate achievement of SDG 3 by 2030. Notwithstanding, addressing health challenges in sub-Saharan Africa requires not one or two interventions, but a package of interventions. The evidence for proven interventions is enormous. What remains is commitment and balancing investments to achieve optimum health outcomes for mothers and newborns.

### Limitations

The study relies on data from Demographic Health Surveys. These household surveys are mainly conducted through verbal interviews with women and heads of households. Because DHS are conducted once in a few years, the interviews mean women have to reflect back on past decisions regarding delivery. While this may be feasible, it is also worth noting that the methodology is subject to recall bias. The definition of urban areas also tend to vary over time since in many countries, national statistical offices tend to define an urban area based on the size of the population and other key characteristics. The population size of towns and cities changes over time thereby affecting comparison of urban areas between surveys. Nevertheless, the strength of this study lies in the use of rich source of national representative Demographic and Health Surveys from 1990 to 2015 to assess changes in health facility delivery within the context of renewed calls at the international level to address existing maternal and child health challenges.

## Conclusion

To achieve the proposed SDG target for maternal mortality ratio (under 70 deaths per 100,000 live births by 2030) in sub-Saharan Africa, more efforts should be made by sub-Saharan African countries. Interventions should focus on addressing challenges related to low levels of maternal education and empowerment, increased access to health facilities as well as narrowing the rural-urban gap. Health system improvements and financing mechanisms should be implemented in line with the framework for universal health coverage. Other countries can learn from Rwanda which has implemented policies to expand maternal and child health services thereby leading to increased health facility births with concomitant reduction in neonatal mortality rates. Reducing the urban-rural gaps in facility births and neonatal mortality remains one of the key strategies effective interventions to improve maternal and child health.

## Appendix

**Table 6:** Observed and expected proportion of health facility delivery by country for 29 sub-Saharan African countries.

Country	Health facility delivery		Ratio (3) = (1) / (2)
	Observed (1)	Expected (2)	
Burkina Faso	24.06	24.13	1.00
Benin	31.77	31.52	1.01
Congo Democratic Republic	33.46	32.87	1.02
Congo Brazzaville	40.05	39.91	1.00
Cote’d’Ivoire	21.55	21.36	1.01
Cameroon	26.21	26.06	1.01
Ethiopia	2.87	3.07	0.93
Gabon	30.63	31.81	0.96
Ghana	28.52	28.47	1.00
Guinea	13.16	12.98	1.01
Comoros	26.5	26.02	1.02
Kenya	24.09	23.9	1.01
Liberia	23.37	23.41	1.00
Lesotho	17.53	17.46	1.00
Madagascar	12.92	12.85	1.01
Malawi	30.11	30.03	1.00
Mali	14.6	14.47	1.01
Mozambique	21.27	21.39	0.99
Nigeria	14.49	14.56	1.00
Niger	9.07	9.05	1.00
Namibia	34.79	34.69	1.00
Rwanda	21.34	21.3	1.00
Sierra Leone	20.06	20.19	0.99
Senegal	27.53	27.53	1.00
Togo	21.7	21.53	1.01
Tanzania	19.31	19.11	1.01
Uganda	19.43	19.28	1.01
Zambia	24.38	24.38	1.00
Zimbabwe	22.39	22.61	0.99

## Abbreviations

ANC: Antenatal Care
DHS: Demographic and Health Survey
MDG: Millennium Development Goals
MMR: Maternal Mortality Ratio
SDG: Sustainable Development Goal
UHC: Universal Health Coverage
